# Economic impact of dengue in Singapore from 2010 to 2020 and the cost-effectiveness of Wolbachia interventions

**DOI:** 10.1371/journal.pgph.0000024

**Published:** 2021-10-13

**Authors:** Stacy Soh, Soon Hoe Ho, Annabel Seah, Janet Ong, Borame Sue Dickens, Ken Wei Tan, Joel Ruihan Koo, Alex R. Cook, Kelvin Bryan Tan, Shuzhen Sim, Lee Ching Ng, Jue Tao Lim

**Affiliations:** 1 Environmental Health Institute, National Environment Agency, Singapore, Singapore; 2 Saw Swee Hock School of Public Health, National University of Singapore, Singapore, Singapore; 3 Ministry of Health, Singapore, Singapore; 4 School of Biological Sciences, Nanyang Technological University, Singapore, Singapore; Pennsylvania State University, UNITED STATES

## Abstract

The release of Wolbachia-infected mosquitoes is a promising disease intervention strategy that aims to control dengue and other arboviral infections. While early field trials and modelling studies suggest promising epidemiological and entomological outcomes, the overall cost effectiveness of the technology is not well studied in a resource rich setting nor under the suppression approach that aims to suppress the wild-type mosquito population through the release of Wolbachia-infected males. We used economical and epidemiological data from 2010 to 2020 to first ascertain the economic and health costs of dengue in Singapore, a high income nation where dengue is hyper-endemic. The hypothetical cost effectiveness of a national Wolbachia suppression program was then evaluated historically from 2010 to 2020. We estimated that the average economic impact of dengue in Singapore from 2010 to 2020 in constant 2010US$ ranged from $1.014 to $2.265 Billion. Using empirically derived disability weights, we estimated a disease burden of 7,645–21,262 DALYs from 2010–2020. Under an assumed steady-state running cost of a national Wolbachia suppression program in Singapore, we conservatively estimate that Wolbachia would cost an estimated $50,453–$100,907 per DALYs averted and would lead to an estimated $329.40 Million saved in economic costs over 2010 to 2020 under 40% intervention efficacy. Wolbachia releases in Singapore are expected to be highly cost-effective and its rollout must be prioritised to reduce the onward spread of dengue.

## 1 Introduction

Increasing urbanization, human population density and climate change has led to an expanded geographical coverage of the primary vector of dengue, *Ae*. *aegypti*, and has resulted in an estimated annual 105 million dengue infections globally [1]. The burden of dengue is high, particularly in the tropics and subtropics where vector breeding conditions are favourable and transmission persists year-round [2–5].

Quantifying the burden of dengue is important for the appropriate allocation of resources among competing public health issues and the evaluation of the cost-effectiveness of interventions. One particular novel intervention for dengue comprises the release of mosquitoes infected with the intracellular bacterium Wolbachia [6]. Mosquitoes infected with Wolbachia are (a) less likely to disseminate a large number of vector-borne diseases, such as dengue [6–8] and (b) can suppress/replace the wild type mosquito population due to cytoplasmic incompatibility [6]. Various countries have ongoing programmes that either aim to achieve stable introgression of Wolbachia into wild type mosquito populations by releasing females, or by suppressing the existing wild-type population through the release of males. These programmes are mostly at the evaluation stage, to ascertain the field efficacy of Wolbachia technologies. As of June 2021, 13 countries have introgression programmes in place, which have been implemented due to its perceived sustainability and low maintenance cost after a period of upfront investment [9, 10]. China [11], the USA [12] and Singapore [13] have suppression-based programmes, which have been implemented due to its perceived compatibility with existing vector control programmes, and greater social acceptability compared to the former approach. A previous study in Yogyarkata, Indonesia has since explored the cost-effectiveness of introgression programmes, taking into account spatio-temporal heterogeneity of the impact of Wolbachia through a modelling approach [14]. However, to the best of the authors’ knowledge, the cost-effectiveness of suppression-based programmes is not yet known.

Singapore presents several unique features of dengue transmission. Singapore is a high income nation with all four dengue serotypes in active circulation and weekly reported case counts above zero for the past 20 years. Due to extensive investments into vector control, the force of infection is estimated to have rapidly declined from the late 1960s to the mid-1990s, after which the force of infection remained stable [15]. However, persistent outbreaks continue to occur in multi-year cycles [16, 17], with the recent 2020 dengue outbreak recording an all-time high of 1,792 weekly cases [18]. This demonstrates the limits of conventional vector control measures such as breeding site elimination and fogging, as well as the importance of evaluating other potential novel vector control strategies, such as Wolbachia.

To ascertain the cost-effectiveness of Wolbachia interventions in Singapore, the respective baseline economic and health costs attributable to dengue requires quantification. National level studies evaluating the economic and health impact of dengue have been carried out in developing countries where dengue is endemic, such as Cambodia, Malaysia and Thailand [19], and also in resource rich contexts such as Puerto Rico, Brunei and Aruba [20–22]. In particular, a previous study in Singapore estimated the economic costs of dengue and the potential cost-effectiveness of vaccines from 2000 to 2009 [23]. However, the economic and health costs as previously estimated from [23] are likely to differ significantly from 2010 to 2020, due to changes in the epidemiological situation of dengue over the past decade. This makes the evaluation of novel interventions, such as Wolbachia, challenging.

To this end, our study aims to triangulate the economic and health burden of dengue in Singapore from 2010 to 2020 and evaluate the retrospective cost-effectiveness of implementing Wolbachia interventions. Sensitivity analysis was then conducted to further understand the possible influences of model parameters to our presented estimates.

## 2 Methods

### 2.1 Data

Reported dengue case counts and deaths stratified by age was collected from the national surveillance system [24] from 2010 to 2020. Under the Infectious Disease Act, reporting of dengue cases to the Ministry of Health is legally mandated in Singapore. The cases notified by registered medical practitioners are collated and published weekly in the Infectious Disease Bulletin by the Ministry of Health [25]. We further obtained the proportion of dengue cases’ ambulatory, hospitalized and emergency department visits to public healthcare institutions from 2010 to 2020. Public hospitals manage around 80% of hospital admissions in Singapore [26].

The epidemic and economic parameters were obtained from previous dengue studies in Singapore [23], literature, official sources and in consultation with the National Environment Agency which is responsible for vector control (Tables 1 and 2).

**Table 1:** Parameters used to estimate economic and health burdens from 2010 to 2020.

Parameter		Value	Source
Proportion of children that require a parent to be absent from work for care giving		0.43	[33]
Proportion of elderly needing to hire a care giver		0.073	[34]
Discount rate for premature deaths productivity lost		0.03	[35]
Transport costs to seek medical care and household members visiting patients^1^		3.7	[36]
Average household services losses per day		35	[37]
Cost of providing primary education per student per day^2^		21.2–36.6	[38, 39]
Cost of providing secondary education per student per day^2^		29.6–48.5	[38, 39]
Average costs per visit (CHAS)^3^, Cost_*C*_		32.8–56.1	MOH
Average costs per visit (Polyclinic)^3^, Cost_*P*_		58.0–74.8	MOH
Average costs per visit (Public Hospitals)^3^		1780.9–3014.0	MOH
Average costs per visit (Emergency Department)^3^		135.3–281.5	MOH
Average productivity loss per absent day of work in individuals from 18 to 64 years^4^		155.4–200.0	[40]
*EF*_*a*_	0–24, age dependent (constant) symptomatic rates^5^	3.8 (1.7–3.6)	[23]
*EF*_*a*_	25–34, age dependent (constant) symptomatic rates^5^	13.1 (3.8–8.2)	[23]
*EF*_*a*_	35–44, age dependent (constant) symptomatic rates^5^	24.3 (6.1–13.4)	[23]
*EF*_*a*_	45–54, age dependent (constant) symptomatic rates^5^	45.3 (11.1–24.2)	[23]
*EF*_*a*_	*>*55, age dependent (constant) symptomatic rates^5^	50 (12.2–26.5)	[23]
Average number of ambulatory visits		4.33	ARDENT project
Duration of disability, reported/unreported cases		4–14	[41]
Hospital average length of stay		3.2–3.7	MOH
*EFh* ^6^		1	MOH

^1^ Average daily ridership and average round trip distance used to calculate weighted average transportation cost. It includes Mass Rapid Transport and Light Rapid Transport systems, bus, and taxi. An average of two family visits per day per inpatient are assumed.

^2^Estimated by dividing the average cost of one student per year by number of schooling days for each year.

^3^Estimated using average bill size per notified dengue patient in respective institution type per year.

^4^Estimated by dividing the household median income by number of calendar years.

^5^Follows [23] by estimating ambulatory expansion factors using serological information in Singapore from 2004 onwards.

^6^Conservatively sets hospitalization expansion factor to 1 by assuming perfect diagnosis.

**Table 2:** Parameters for DALYs and Wolbachia programme cost-effectiveness computation.

Parameter	Value	Source
Disability weight for symptomatic cases of DF from WHO, (literature), *D*	0.211 (0.81)	[42, 43]
Disability weight for symptomatic cases of DHF from WHO, (literature), *D*	0.5 (0.85)	[43, 44]
Mean disability weight for symptomatic ambulatory, (hospitalized) children cases, *D*	0.37 (0.52)	[45]
Mean disability weight for symptomatic ambulatory, (hospitalized) adult cases, *D*	0.42 (0.53)	[45]
Social discount rate for DALYs calculations, *r*	0.03	[35, 46]
Age-weighting correction constant, *C*	0.16243	[46]
Parameter of the age-weighting function, *β*	0.04	[46]
Duration of disability in reported cases	10.4	[27, 47], ARDENT project
Duration of disability in unreported cases	4	[41]
Duration of disability in DHF cases	14	[22, 48]
Discount rate for premature deaths productivity lost	0.03	[35]
Median life expectancy of a Singaporean	85	[49]
Wolbachia steady-state cost per year^1^ (Mn, SGD)	40	NEA
Wolbachia steady-state cost per year^2^ (Mn, 2010USD)	22.7	Derived from the NEA estimate
% estimated reduction in dengue from Wolbachia programme^3^	40%– 80%	[50]

^1^Yearly annual average cost of a national Wolbachia programme. This figure comprises renovation and equipment cost for mosquito production facilities; operating costs such as facility rental and maintenance, utilities, and consumables for mosquito production; manpower costs for production and release, and cost of community engagement initiatives.

^2^We derived the 2010USD cost using the mean exchange rate from 2010 to 2020 and the 2010 price level from the estimate in ^1^.

^3^Estimated in sites undergoing Wolbachia suppression programme trials which already have baseline levels of existing vector control measures.

### 2.2 Accounting for underreporting

We followed Carrasco 2011 [23] and accounted for underreporting by using expansion factors [27] (EF) to scale the reported cases. Expansion factors were only applied to ambulatory cases where *EF*_*i*_ denotes the expansion factor applied to age group *i* and were obtained from [28]. Following Carrasco 2011 [23], we considered two scenarios for deriving the expansion factors across age groups, which were (i) age-dependent symptomatic rate [29] and (ii) a constant range of symptomatic rates across age groups [30, 31]. No expansion factor was applied to hospitalized cases, as all suspected cases of dengue fever/dengue haemorrhagic fever were tested to confirm the diagnosis on admission to secondary care institutions in Singapore.

### 2.3 Direct costs

We considered both medical and non-medical direct costs. Direct medical costs were calculated for hospitalized, emergency and ambulatory cases. Daily hospitalization costs were available from 2010 to 2020 at the individual level for public hospitals, while the unit cost of ambulatory dengue cases was taken from a weighted average of private primary care clinics under the Community Health Assist Scheme [32] and polyclinics. These respectively represent subsidized private and public medical institutions providing primary care for acute/chronic illnesses. The unit cost was determined by first obtaining the total cost attributable to reported ambulatory dengue cases from both the Community Health Assist Scheme Cost_*C*_*N*_*C*_ [32] and polyclinic cases Cost_*P*_ *N*_*P*_. This was then divided by the total number of reported ambulatory dengue cases from these institutions *N*_*C*_ + *N*_*P*_:

$$Cost_{Amb}=\frac{Cost_{C}N_{C}+Cost_{P}N_{P}}{N_{c}+N_{p}}$$ (1)

The total costs of ambulatory cases were obtained by multiplying the expected number of visits per case by the unit cost of each visit (Table 1). The cost breakdown for each ambulatory visit included treatment and consultation costs. Non-medical direct costs included individual and family transport costs for visitations (Table 1).

### 2.4 Indirect costs

Indirect costs were expressed per unspecified day and included the reduction of work productivity, the reduction of household services, the loss of schooling, and the increased need for caregivers. We followed standardized guidelines on estimating work productivity losses by (1) the human capital [51] and (2) friction cost method [52]. The human capital method values lost time or premature death using the individual’s gross earnings, as approximated by median household income per year [23]. The friction cost method acknowledges that job absenteeism or death leading to productivity losses may be temporarily offset by colleagues or by hiring new labour [52]. Under the friction cost method, loss of productivity occurs only during an assumed friction time period of 30 days for fatalities and lasts as long as the symptoms in non-fatal cases. Productivity losses under the friction cost method were offset according to the elasticity of annual labour time versus labour productivity (Table 2). Friction costs *FC* were then calculated by multiplying the length of the friction period *t*_2_ –*t*_1_ with the expected average gross earnings $W_{t_{1}:t_{2}}$ in the period and the elasticity of annual labour time versus labour productivity *ϵ*:

$$FC=\left(t_{2}-t_{1}\right)\times W_{t_{1}:t_{2}}\times \epsilon$$

In Singapore, primary and secondary school education is compulsory for children and adolescents aged 6 to 12 and 13 to 16 respectively. We assumed that individuals within these age ranges incur costs from school days lost, and these were estimated as the cost per primary/secondary school student per schooling day.

We also estimated the impact of dengue illness on household services, which were not paid for but represent important economic activity (e.g. cleaning, cooking, care-taking of other household members) (Table 1) [37]. We attributed the losses of household services not only to the working population but also the young and the elderly for the duration of disability, at different proportions [37].

We assumed that symptomatic children and elderly caused further parental/child job absenteeism from caretaking. For a proportion of the elderly, outpatients were assumed to require an additional caregiver (Table 1).

### 2.5 DALYs estimation

We employed two sets of disability weights: (1) age-dependent disability weights—based on whether an individual is a child or adult, as estimated in [45], and (2) constant disability weights—based on the severity of illness, i.e dengue fever or dengue haemorrhagic fever [42–44]. A disability weight of 1 was used for premature death (i.e, below the median life expectancy of a Singaporean in 2020).

DALYs lost by each case were calculated using:

$$-\frac{DCe^{-\beta \alpha^{2}}}{\beta +r}\left[e^{-\left(\beta +r\right)L}\left(1+\left(\beta +r\right)\left(L+\alpha \right)\right)-\left(1+\left(\beta +r\right)\alpha \right)\right]$$

where *D* is the disability weight; *r* is the social discount rate; *α* is the age of the individual at the onset of symptoms; *L* is the duration of the disability or the years of life lost due to premature death expressed in years; *C* is the age-weighting correction constant; and *β* is the parameter from the age-weighting function. The age-weighting function represents the value of life at different ages [23]. Heuristically, (2) could be viewed as the DALYs lost for a disease episode weighted against the duration of disability and the age of the individual experiencing that disease episode.

### 2.6 Cost-effectiveness of Wolbachia

We conservatively estimated the cost-effectiveness of Wolbachia interventions using epidemiological information from Singapore-specific field trials [50], where vector control activity was present as a baseline in these locales. Specifically, a reduction of dengue incidence of 71% (43%–87%) to 88% (57%–99%) under Wolbachia-based incompatible insect technique was used to evaluate the cost-effectiveness of Wolbachia. The scaled-up, steady state cost of implementation Wolbachia-based intervention for the whole of Singapore was estimated to be around SG$40Mn (2010US$22.7Mn). We obtained the counterfactual cost and health burden reduction for 2010 to 2020 by (1) deflating the observed ambulatory case, hospitalization and death burden using the range of percentage reductions attributable to Wolbachia-based intervention by the same proportion (2) calculating the counterfactual economic cost and health burdens under this scenario.

The economic cost savings due to Wolbachia were then obtained by comparing the reduction in economic costs versus the projected steady state cost of Wolbachia operations in Singapore. Cost-effectiveness was determined by comparing the reduction in DALYs per 100,000 per dollar invested due to Wolbachia with overall thresholds for the cost effectiveness of interventions as determined from literature:

$$\$\;per\;DALY\;averted=\frac{Cost\;of\;intervention\;under\;steady\;state}{Estimated\;DALYs\;averted\;under\;intervention}$$

We incorporated the uncertainty in the economic and health cost estimation by stochastic simulation. Namely, we simulated from a uniform distribution with the minimum and maximum parameters being the range of values each parameter possesses. For each unique draw, we recomputed all components of the economic and health cost. This was conducted 1000 times, and we obtained their 95% uncertainty interval (UI) by taking the 95% quantiles of the values of interest in the simulated samples. All dollar costs were expressed in 2010US dollars unless otherwise specified.

## 3 Results

### 3.1 Economic and disease burden

**Fig 1:**
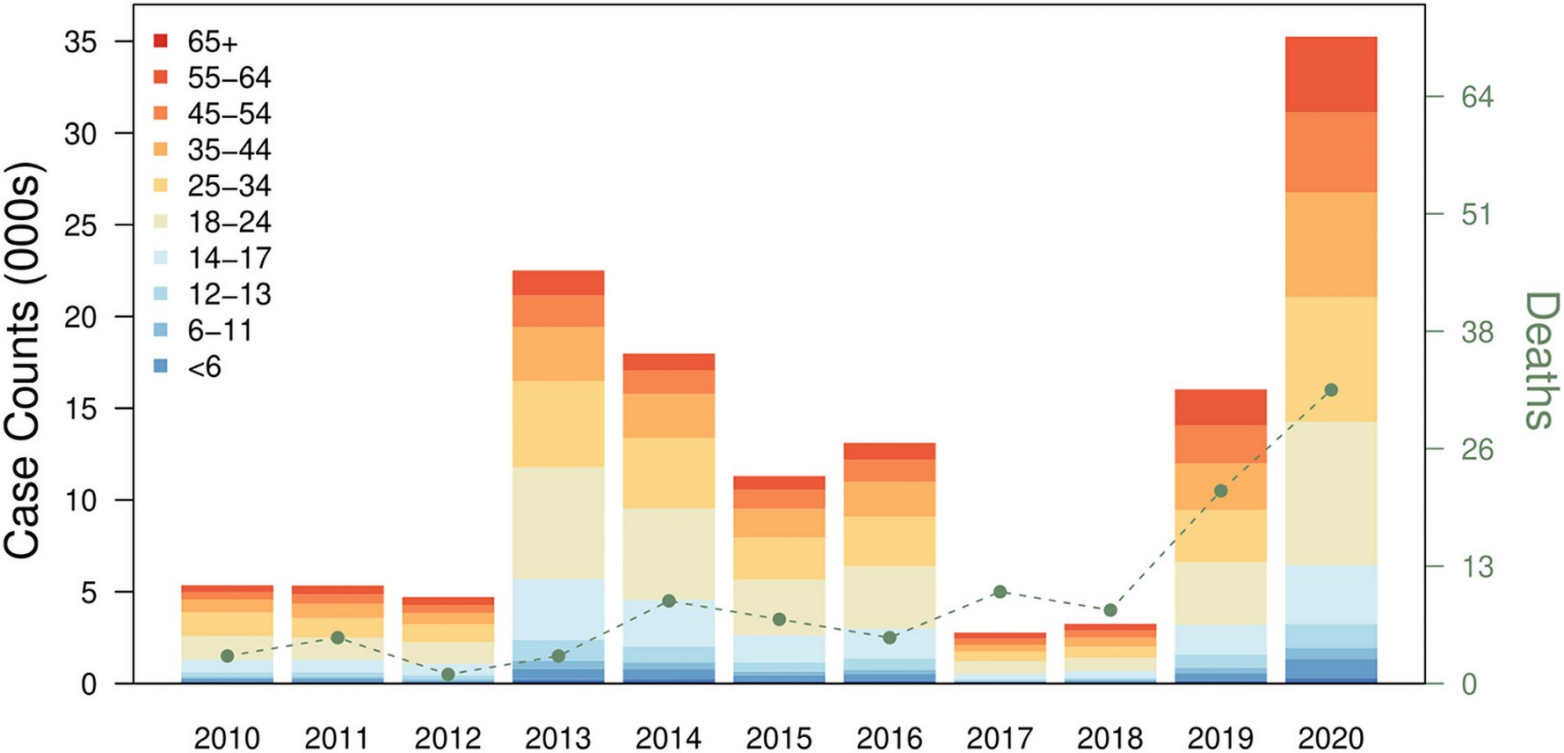
Dengue reported case counts and deaths from 2010 to 2020. The box plots represent case counts stratified by age groups and the lines represent reported deaths due to dengue illness.

The annual reported dengue case counts from 2010 to 2020 ranged from 2,767 in 2017 to 35,315 in 2020, with the largest number of individuals reported to have dengue in the 25–34 age group, followed by the 34–44 and 45–54 age groups (Fig 1). Comparatively, the burden was relatively lower in the paediatric and adolescent groups, with individuals aged 17 and below comprising approximately 10% of reported dengue case counts. The number of deaths were relatively stable from 2010 to 2018, ranging from 0 to 10, but increased considerably to almost twice the amount to 21 and 32 for 2019 and 2020 respectively (Fig 1).

**Fig 2:**
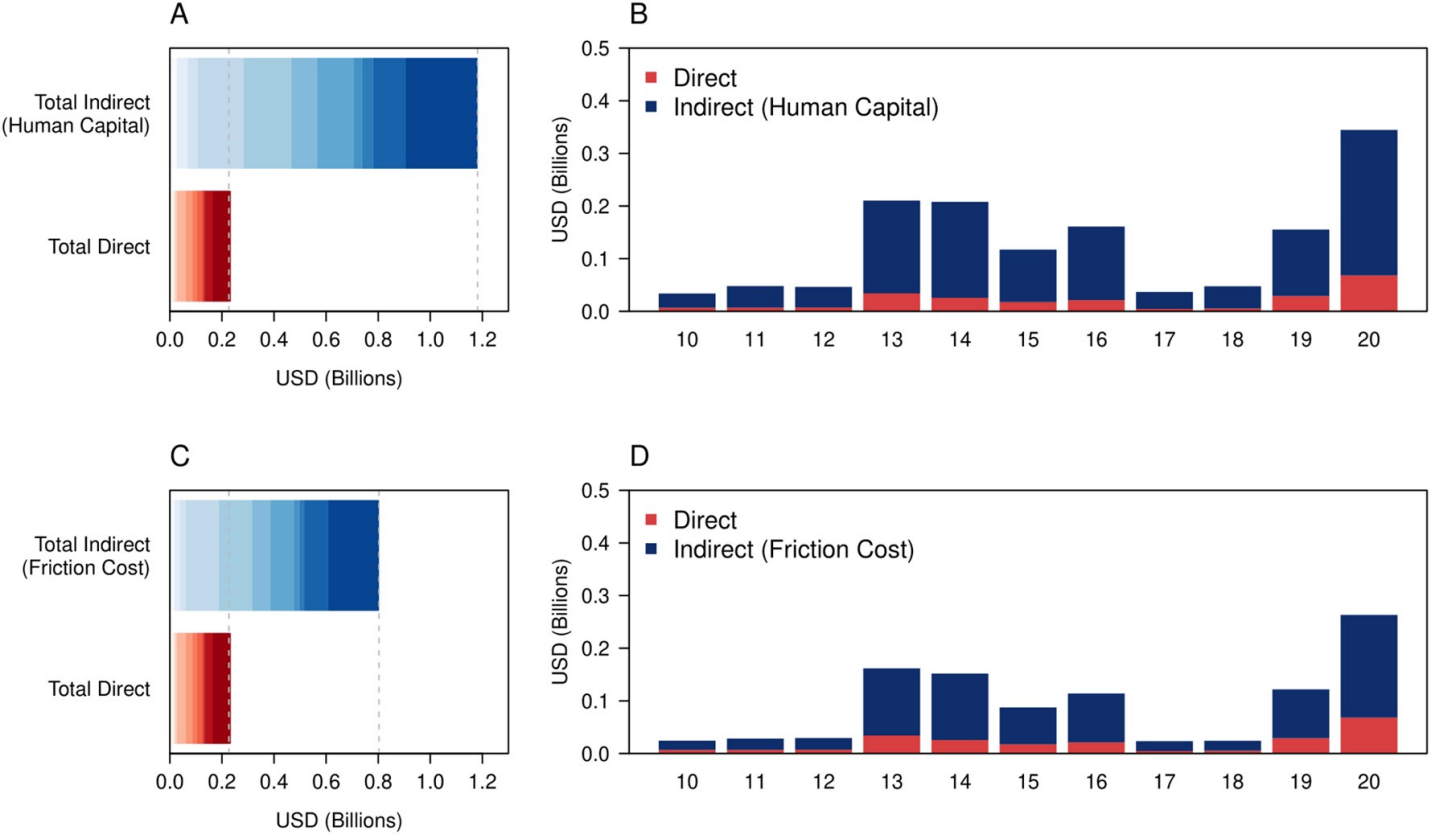
A) Breakdown of the economic costs under human capital method by indirect and direct costs. The darker shades indicate more recent years B) Breakdown of economic costs under human capital method by years C) Breakdown of economic costs under friction cost method by indirect and direct costs. The darker shades indicate more recent years D) Breakdown of economic costs under friction cost method by years.

Using the more conservative friction cost method with constant symptomatic rates, we estimated that the total economic cost of dengue across 2010 to 2020 was 2010US$1.014 Billion (Bn) (Fig 2, 95% UI: $0.796 –$ 1.276). The total cost increased to 2010US$2.265 Billion (Bn) (Fig 2, 95% UI: $1.927 –$2.618) after incorporating age-dependent symptomatic rates. The human capital method resulted in significantly higher costs, with the total economic cost estimated to be at 2010US$1.401 Billion (Bn) (Fig 2, 95% UI: $1.182 –$1.628) and 2010US$3.003 Billion under the constant and age-dependent symptomatic rates respectively, driven primarily by the increased costs of deaths under this method. Between using the constant symptomatic rate and age-dependent symptomatic rate assumptions, the higher costs under the age-dependent symptomatic rate assumption were due to the higher number of ambulatory cases caused by larger expansion factors. The number of deaths and hospitalizations did not vary. A large proportion of costs under all scenarios was also driven by the indirect costs, especially work productivity loss. For example, under the friction cost method with constant symptomatic rates, 77.704% of the total costs were due to the indirect costs, with work productivity loss from illness and death accounting for 74.076% of the total costs.

The estimated economic costs from dengue per year also followed the annual reported dengue case counts closely, as opposed to deaths. Taking the friction cost method with constant symptomatic rates, the estimated economic costs were relatively low in years where reported dengue case counts were suppressed, such as 2017 (Fig 2A and 2B: 2010US$0.023, 95% UI $0.018 –$0.029 Bn) and high when reported dengue case counts were elevated, such as 2020 (Fig 2A and 2B: 2010US$0.262, 95% UI $0.207 –$0.324 Bn).

**Fig 3:**
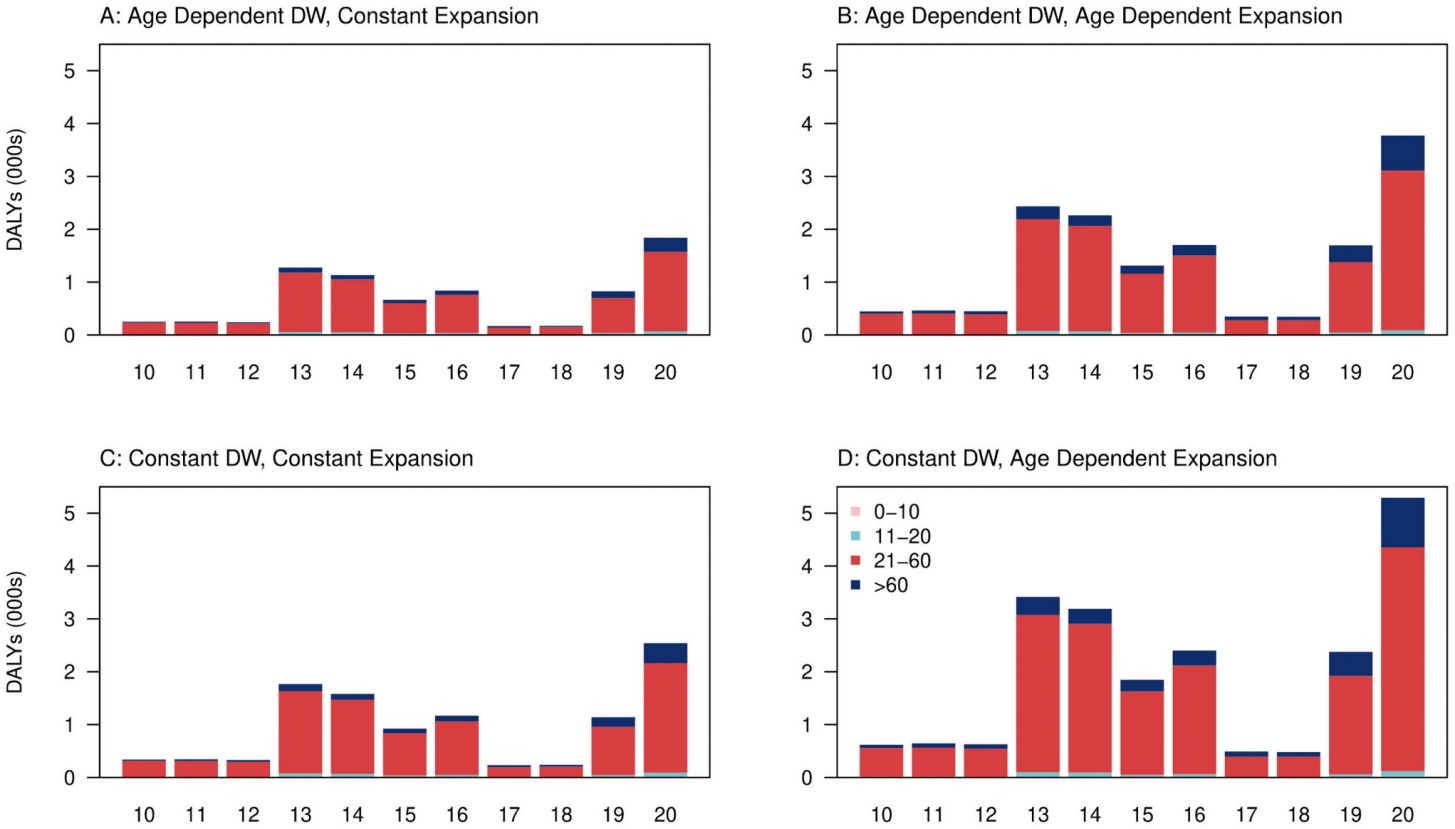
Breakdown of DALYs (in thousands) per year from 2010 to 2020 under A) age dependent disability weights (DW), constant expansion factors B) age dependent disability weights, age dependent expansion factors C) constant disability weights, constant expansion factors D) constant disability weights, age dependent expansion factors.

Using a constant expansion factor, we estimated the overall DALYs over 2010 to 2020 to be 10,559 (Fig 3B, 95% UI: 8,148–13025) when using constant symptomatic rates disability weights and 7,645 (Fig 3A, 95% UI: 6,837–8,497) when using age-dependent symptomatic rates disability weights. Using age-dependent expansion factors, we estimated the average DALYs to be 21,262 (Fig 3D, 95% UI: 16,272–26,433) when using constant symptomatic rates disability weights, and 15,214 (Fig 3C, 95% UI: 13,593–16,909) when using age-dependent symptomatic rates disability weights.

### 3.2 Cost-effectiveness of Wolbachia interventions

Assuming steady state costs of SG$40 Million (Mn) (2010US$22.7Mn) per year, Wolbachia was found to be overall cost effective under 40% intervention effectiveness or above. At 40% intervention effectiveness, we found that over 2010US$329.40Mn would have been averted in economic costs from 2010 to 2020. As the assumed intervention effectiveness of Wolbachia increases from 40% to 80%, the estimated cost averted would also have increased to 2010US$658.79Mn from 2010 to 2020 (Table 3).

**Table 3:** Estimated economic and health costs and hypothetical burden averted from Wolbachia interventions.

Year	2010	2011	2012	2013	2014	2015	2016	2017	2018	2019	2020
Estimated economic cost (Mn, USD2010)^1^	24.15	28.20	29.25	160.64	150.89	86.94	113.17	23.22	23.90	121.04	261.68
Estimated economic cost (Mn, SGD)^2^	32.84	36.60	38.40	214.85	208.87	129.83	171.79	35.89	37.11	192.59	429.72
Case Prevention (40% Efficacy)^3^	1,712	1,708	1,508	7,203	5,753	3,618	4,197	889	1,041	5,130	11,282
Incidence Prevention (40% Efficacy)^4^	4,362	4,529	4,387	21,844	19,793	11,910	15,413	3,466	3,441	17,392	38,255
Estimated DALYs^5^	250	252	242	1282	1139	667	842	168	173	831	1851
US$000s per DALYs averted (40% Efficacy)^5^	285	282	295	56	63	107	85	424	412	86	39
SG$000s per DALYs averted (40% Efficacy)^6^	502	498	519	98	110	188	149	749	723	151	68
	Costs Averted (Mn, 2010USD)										
40% efficacy^7^	7.76	9.06	9.40	51.677	48.55	27.9	36.40	7.47	7.68	38.91	84.11
50% efficacy^7^	9.70	11.33	11.76	64.58	60.68	34.96	45.50	9.33	9.61	48.64	105.14
60% efficacy^7^	11.64	13.60	14.11	77.50	72.82	41.95	54.60	11.20	11.53	58.36	126.17
70% efficacy^7^	13.58	15.86	16.46	90.42	84.95	48.94	63.71	13.07	13.45	68.09	147.20
80% efficacy^7^	15.52	18.13	18.81	103.33	97.09	55.93	72.81	41.93	15.37	77.82	168.22
90% efficacy^7^	17.46	20.40	21.16	116.25	109.23	62.92	81.91	16.80	17.29	87.54	189.25

^1^Direct and indirect economic costs attributable to dengue from 2010 to 2020 under the friction cost method and constant symptomatic rate expansion factors in millions (Mn) 2010USD.

^2^Direct and indirect economic costs attributable to dengue from 2010 to 2020 under the friction cost method and constant symptomatic rate expansion factors in nominal Singapore dollars (SGD).

^3^Hypothetical dengue cases averted from the national implementation of Wolbachia interventions.

^4^Hypothetical dengue incidence averted from the national implementation of Wolbachia interventions, as calculated under constant symptomatic rate expansion factors.

^5^ DALYs were computed using age dependent disability weights, with constant symptomatic rate expansion factors.

^6^Hypothetical $ per DALYs averted, assuming steady state cost of 40Mn SGD a year in 2020 for national Wolbachia programme and intervention efficacy of 40%. DALYs were computed using age dependent disability weights, with constant symptomatic rate expansion factors. $ per DALYs averted were reported in nominal SGD here.

^7^Hypothetical economic costs averted assuming percentage reductions in dengue cases from national implementation of Wolbachia interventions in 2010USD.

In terms of health burden, using age dependent disability weights and constant expansion factors as representative assumptions for DALYs computation, we estimated that Wolbachia interventions would have cost per DALYs averted at 40% intervention effectiveness at 2010US$100,907, while cost per DALYs averted would have decreased to 2010US$50,453 as intervention effectiveness increases to 80% (Table 3).

### 3.3 Sensitivity analysis

We evaluated the sensitivity of the mean estimated economic and health costs to variations in our model parameters. We performed univariate sensitivity analysis where each parameter was increased by 25% to evaluate their respective importance. Sensitivity analysis demonstrated that the economic burden was most sensitive to the (1) age-dependent/constant symptomatic rate expansion factors for ambulatory cases, (2) number of ambulatory visit days and (3) number of days absent from ambulatory visits and (4) either an increase in hospital expansion factors or the elasticity of labour product. This demonstrates that economic costs were driven primarily by ambulatory cases, through job absenteeism and a lost in labour productivity. Inflating these parameters led to around a 2.8%– 24.4% increase in total economic costs from the baseline levels as estimated from the preceding sections.

Health costs were most sensitive to the (1) duration of disability for children (2) ambulatory expansion factors and (3) duration of disability for unreported dengue fever. In particular, DALYs estimates were far more sensitive under inflated parameter inputs under the assumption of constant disability weights and age dependent symptomatic rate ambulatory expansion factors, with DALYs inflated to 275% their original values when the duration of disability for children was increased 25%. In comparison, changing the parameters under the assumption of age dependent disability weights and constant symptomatic rate ambulatory expansion factors, which our primary results were reported with, changed the DALYs estimates by 70% when ambulatory expansion factors were inflated. The supplementary information contains the full ranking of importance among the parameters used to compute economic and health burdens.

## 4 Discussion

Under our most conservative assumptions, we estimated the total direct and indirect economic cost of dengue to be around 2010US$1.014 billion (Bn) (Fig 2C, 95% UI: $0.796 –$ 1.276) from 2010 to 2020, which was on average around 2010US$90 million (Mn) per year. Removing the vector-control costs from previous economic cost estimates for dengue in Singapore from 2000 to 2009 ($0.4 –$0.65Bn) [23], the yearly economic costs of dengue is estimated to be almost twice as high in 2010 to 2020 in real terms. Similarly, our yearly estimate of dengue cost was nearly twice that of Shepard et al.’s estimate of approximately $52 Mn [53]. Our parameters however differ from the study by Carrasco and colleagues [23] and Shepard and colleagues [53] in several ways, which make our economic burden estimates far more conservative. Hospital expansion factors were assumed to be unity in our study, due to the improved diagnostic capacities in these institutions over the years [54]. Furthermore, our study used median income instead of gross domestic product per capita to remove income outliers. Further stratification of the healthcare costs to exclusively primary and secondary care public institutions, where costs are heavily controlled, was also likely to have reduced our estimates further.

Looking specifically at the individual year breakdowns of economic costs, we found that the largest burden was attributed to 2020, standing at 2010US$0.262 Bn. The second largest economic cost attributable to dengue was 2010US$0.161 Bn in 2013, which was around 38.5% lower compared to 2020. There are several key reasons which may have led to the excess cost in 2020, which include the increased dengue transmission in the general community attributed to non-pharmaceutical interventions motivated by SARS-CoV-2 [55] as well as the serotype switch to DENV-3, a serotype which the Singapore population has low immunity to [15, 56, 57]. However, average economic costs only fell to 2010US$76 Mn per year from 2010–2019, demonstrating that dengue burden has indeed increased compared to 2000–2009 in real terms despite the exclusion of 2020 as an outlier year. The increasing human population density, population and urbanicity over the years has led to increased dengue transmission potential [58] due to expanding niches of the *Ae*. *aegypti* vector. Paradoxically, the success of the national vector control programme has redistributed the predominant age of reported dengue cases to the working adult group, while it remains primarily a pediatric illness in other dengue endemic nations [15]. Despite initiatives to improve the triaging of individuals presenting symptoms of dengue illness to lower cost primary care institutions [59], high employment rates coupled with increasing median incomes in the working age group has resulted in a sizeable contribution to indirect economic costs and an increase in dengue burden over the past decade. Our results reveal the increasing difficulty in controlling the economic costs of dengue, despite having a world leading vector control programme. This study also demonstrates the importance of developing novel, cost-effective vector control tools to stem the spread of dengue virus.

By setting the baseline steady state cost of Wolbachia suppression programmes in Singapore to be SG$40Mn (2010US$22.7Mn) per year, we estimated that the cost savings from Wolbachia interventions will on average be achieved if the interventions result in a 40% reduction in dengue case counts across all groups. Estimates from the field studies in Singapore demonstrate that an estimated 40–80% reduction in dengue case counts over an extended period of time can be achieved by Wolbachia–demonstrating conservatively that the Wolbachia interventions will lead to cost savings if the suppression programme is implemented at a national level.

We further demonstrated conservatively that Wolbachia interventions will lead to 2010US $50,453–100,907 per DALYs averted, which encompasses the average intervention efficacy of $69,499 per DALYs averted for very high HDI countries [60]. However, costs are far higher compared to the currently available estimate of $1500 per DALYs averted under the replacement programme in Yogyakarta [14]. There are several competing factors driving the disparities in the cost-effectiveness of the Wolbachia programme: (1) labour and equipment costs in Singapore are far higher than Indonesia, (2) the replacement approach includes a large number of one off costs mostly related to releases that occur in the initial year of intervention, whereas the suppression approach relies on the assumption that constant release is necessary to maintain low wild-type mosquito populations, (3) our estimates of Wolbachia effectiveness, and by extension its cost-effectiveness come from field data/trials rather than model-based extrapolations of effectiveness based on laboratory data (4) Singapore presents an overall different epidemiological situation, with a drastically higher population density at 7,810 persons per square kilometre [61] compared to urban areas included in [14]. In comparison, the densest release area was the Jakarta metropolitan area [14] with only 4,383 persons per square kilometre in 2021. Higher population densities compressed into a smaller land area would signify an increased efficacy for Wolbachia due to the larger number of individuals covered by the intervention per release [14], leading to more efficient reductions in the eventual health burden in terms of DALYs and lastly, (5) the lowered herd immunity in the adult group compared to other nations in South-east Asia. Recent serological studies indicate that 90% of children in Indonesia have been exposed to dengue by the age of 12 years old [62], while the overall seroprevalence amongst children aged 1–17 was 10.4% in Singapore and around 13.8%– 35.6% in the 15–39 age group [63, 64]. The predominantly pediatric nature of dengue illness in Indonesia, as studied by Brady and colleagues [14], indicates that the estimated DALYs are far lower compared to Singapore where dengue illness mainly affects the working age groups. Future work should however ascertain the long term burden and cost reductions attributable to Wolbachia taking into account changes in herd immunity, vector competence and demographics.

Several other field trials have been conducted to estimate the cost-effectiveness (per DALY averted) for alternative vector control measures, such as community participation campaigns ($3953/DALY) [65] and ultra-low volume spraying ($4472/DALY) in Colima, Mexico [65], and larviciding campaigns in Cambodia ($313/DALY saved) [66]. Short-term field trials are unlikely to account for several confounders such as herd immunity, antibody dependent enhancement, across-strain interactions, host/vector population dynamics and climate variations. The model based approaches can account for long-term dynamics, but are dependent on model specifications and cannot be ex-ante assessed for theoretical validity. These endeavours have yielded modelled efficacies for larval control ($615-1267/DALY) [14, 67], and more generic packages of vector control ($679-1907/DALY) [14, 68]. Our approach ascertains the cost-effectiveness using a hybrid approach, by calibrating a model-based costing framework using Wolbachia field trial data and estimating the intervention efficacy based on historically observed epidemiological information. Conservatively, these results demonstrate that Wolbachia, under the suppression approach, is possibly one of the more cost effective tools available as an intervention for dengue relative to alternative methods for vector control.

However, a key drawback of our hybrid approach is that we were only able to estimate the cost-effectiveness of Wolbachia above and beyond the baseline the vector control efforts, rather than the independent effect of the Wolbachia interventions. Future work is needed to understand the independent and interactive impact of Wolbachia interventions under the suppression approach and vector control efforts. Furthermore, recent clinical trials signal the promise of newly developed dengue vaccines such as TAK-003, though the field efficacy and cost-effectiveness is not yet available. Future work should explore how these novel pharmaceutical interventions work independently and together with recently developed vector control tools such as Wolbachia.

Several key limitations arise from our study. First, the potential impact of Wolbachia interventions on other vector-borne diseases was not considered. Singapore had experienced previous outbreaks of Chikugunya and Zika [69, 70], and Wolbachia has been demonstrated to have protective effects against these pathogens through vector suppression. Thus, the cost effectiveness of Wolbachia in this respect is potentially an underestimate. Second, the cost of the Wolbachia interventions was assumed to be a steady state cost, while actual costs may differ depending on programme implementation. Third, our evaluation of the intervention cost-effectiveness was retrospective in nature. Future cost-effectiveness may be confounded by changes in population size, immunity, climate change and changes in environmental suitability for the vector. Fourth, we used estimates of the within-site intervention efficacy to determine the cost-effectiveness of the Wolbachia programme and did not consider potential spillover treatment effects to other areas. Our estimates for the Wolbachia intervention efficacy are likely to be an underestimate of the total intervention efficacy should the programme be implemented nationally. Consequently, the programme cost-effectiveness is likely to be underestimated. Lastly, while the economic and health burden estimates were obtained where possible by Singapore specific parameters, some parameters may not be representative of local social dynamics, such as the proportion of elderly requiring a care giver. Future burden estimates should update these figures with Singapore specific parameters.

## 5 Conclusion

Our analysis leveraged on the comprehensive dengue surveillance system in Singapore. Using detailed and nationally representative cost information, we ascertained the economic and health burden of dengue in Singapore from 2010 to 2020. By conducting sensitivity analysis and deriving multiple estimates from a large set of assumptions and parameters, we were able to arrive at conservative estimates of the economic and health burden of dengue. Underlying uncertainty was incorporated through simulation where possible, and we were able to arrive at a range of values for each estimate. As a consequence, the cost-effectiveness of Wolbachia was meaningfully estimated under a hypothetical scenario where it was implemented nationally the past decade. Our results demonstrate the potential for Wolbachia under a suppression-based approach to yield significant economic and health costs savings even in a high income setting.

## Supporting information

S1 Appendix Appendix containing additional details on results. (PDF)

S1 Checklist Consolidated Health Economic Evaluation Reporting Standards—CHEERS checklist. (PDF)

## References

[1] Cattarino L, Rodriguez-Barraquer I, Imai N, Cummings DAT, Ferguson NM. Mapping global variation in dengue transmission intensity. Sci Transl Med. 2020 Jan 29;12(528):eaax4144. doi: 10.1126/scitranslmed.aax4144 31996463

[2] Dickens BL, Sun H, Jit M, Cook AR, Carrasco LR. Determining environmental and anthropogenic factors which explain the global distribution of *Aedes aegypti* and *Ae*. *albopictus*. BMJ Glob Health. 2018 Sep;3(4):e000801. doi: 10.1136/bmjgh-2018-000801 30233829 PMC6135425

[3] van Panhuis WG, Choisy M, Xiong X, Chok NS, Akarasewi P, Iamsirithaworn S, et al. Region-wide synchrony and traveling waves of dengue across eight countries in Southeast Asia. Proc Natl Acad Sci. 2015 Oct 20;112(42):13069–74. doi: 10.1073/pnas.1501375112 26438851 PMC4620875

[4] Ooi E-E, Gubler DJ. Dengue in Southeast Asia: epidemiological characteristics and strategic challenges in disease prevention. Cad Saúde Pública. 2009;25(suppl 1):S115–24. doi: 10.1590/s0102-311x2009001300011 19287856

[5] Scott TW, Clark GG, Lorenz LH, Amerasinghe PH, Reiter P, Edman JD. Detection of Multiple Blood Feeding in Aedes aegypti (Diptera: Culicidae) During a Single Gonotrophic Cycle Using a Histologic Technique. J Med Entomol. 1993 Jan 1;30(1):94–9. doi: 10.1093/jmedent/30.1.94 8433350

[6] Walker T, Johnson PH, Moreira LA, Iturbe-Ormaetxe I, Frentiu FD, McMeniman CJ, et al. The wMel Wolbachia strain blocks dengue and invades caged Aedes aegypti populations. Nature. 2011 Aug;476(7361):450–3. doi: 10.1038/nature10355 21866159

[7] Aliota MT, Peinado SA, Velez ID, Osorio JE. The wMel strain of Wolbachia Reduces Transmission of Zika virus by Aedes aegypti. Sci Rep. 2016 Sep;6(1):28792. doi: 10.1038/srep28792 27364935 PMC4929456

[8] van den Hurk AF, Hall-Mendelin S, Pyke AT, Frentiu FD, McElroy K, Day A, et al. Impact of Wolbachia on Infection with Chikungunya and Yellow Fever Viruses in the Mosquito Vector Aedes aegypti. Turell MJ, editor. PLoS Negl Trop Dis. 2012 Nov 1;6(11):e1892. doi: 10.1371/journal.pntd.0001892 23133693 PMC3486898

[9] World Mosquito Program. World Mosquito Program [Internet]. [cited 2021 Sep 28]. https://www.worldmosquitoprogram.org

[10] Nazni WA, Hoffmann AA, Noor Afizah A, Cheong YL, Mancini MV, Golding N, et al. Establishment of Wolbachia strain wAlbB in Malaysian populations of Aedes aegypti for dengue control [Internet]. Microbiology; 2019 Sep [cited 2021 Sep 28]. http://biorxiv.org/lookup/doi/10.1101/775965 10.1016/j.cub.2019.11.007 PMC6926472 31761702

[11] Zheng X, Zhang D, Li Y, Yang C, Wu Y, Liang X, et al. Incompatible and sterile insect techniques combined eliminate mosquitoes. Nature. 2019 Aug 1;572(7767):56–61. doi: 10.1038/s41586-019-1407-9 31316207

[12] Mains JW, Kelly PH, Dobson KL, Petrie WD, Dobson SL. Localized Control of Aedes aegypti (Diptera: Culicidae) in Miami, FL, via Inundative Releases of Wolbachia-Infected Male Mosquitoes. J Med Entomol. 2019 Sep 3;56(5):1296–303. doi: 10.1093/jme/tjz051 31008514

[13] National Environment Agency. Wolbachia-Aedes Mosquito Suppression Strategy [Internet]. [cited 2021 Sep 28]. https://www.nea.gov.sg/corporate-functions/resources/research/wolbachia-aedes-mosquito-suppression-strategy

[14] Brady OJ, Kharisma DD, Wilastonegoro NN, O’Reilly KM, Hendrickx E, Bastos LS, et al. The cost-effectiveness of controlling dengue in Indonesia using wMel Wolbachia released at scale: a modelling study. BMC Med. 2020 Dec;18(1):186. doi: 10.1186/s12916-020-01638-2 32641039 PMC7346418

[15] Tan LK, Low SL, Sun H, Shi Y, Liu L, Lam S, et al. Force of Infection and True Infection Rate of Dengue in Singapore: Implications for Dengue Control and Management. Am J Epidemiol. 2019 Aug 1;188(8):1529–38. doi: 10.1093/aje/kwz110 31062837 PMC6670050

[16] Lim JT, Dickens BS, Haoyang S, Ching NL, Cook AR. Inference on dengue epidemics with Bayesian regime switching models. Kouyos RD, editor. PLOS Comput Biol. 2020 May 1;16(5):e1007839. doi: 10.1371/journal.pcbi.1007839 32357146 PMC7219790

[17] Lim JT, Dickens BL, Ong J, Aik J, Lee VJ, Cook AR, et al. Decreased dengue transmission in migrant worker populations in Singapore attributable to SARS-CoV-2 quarantine measures. J Travel Med. 2021 Feb 23;28(2):taaa228. doi: 10.1093/jtm/taaa228 33274384 PMC7798931

[18] National Environment Agency. Weekly Dengue Cases At The Lowest In 2020 As Community Rallied To Fight Dengue In Historic Outbreak Year [Internet]. [cited 2021 Sep 28]. https://www.nea.gov.sg/media/news/news/index/weekly-dengue-cases-at-the-lowest-in-2020-as-community-rallied-to-fight-dengue-in-historic-outbreak-year

[19] Suaya JA, Shepard DS, Siqueira JB, Martelli CT, Lum LCS, Tan LH, et al. Cost of dengue cases in eight countries in the Americas and Asia: a prospective study. Am J Trop Med Hyg. 2009 May;80(5):846–55. 19407136

[20] Halasa YA, Shepard DS, Zeng W. Economic Cost of Dengue in Puerto Rico. Am J Trop Med Hyg. 2012 May 1;86(5):745–52. doi: 10.4269/ajtmh.2012.11-0784 22556069 PMC3335675

[21] Shepard DS, Undurraga EA, Halasa YA. Economic and Disease Burden of Dengue in Southeast Asia. Gubler DJ, editor. PLoS Negl Trop Dis. 2013 Feb 21;7(2):e2055. doi: 10.1371/journal.pntd.0002055 23437406 PMC3578748

[22] Shepard DS, Coudeville L, Halasa YA, Zambrano B, Dayan GH. Economic Impact of Dengue Illness in the Americas. Am J Trop Med Hyg. 2011 Feb 4;84(2):200–7. doi: 10.4269/ajtmh.2011.10-0503 21292885 PMC3029168

[23] Carrasco LR, Lee LK, Lee VJ, Ooi EE, Shepard DS, Thein TL, et al. Economic Impact of Dengue Illness and the Cost-Effectiveness of Future Vaccination Programs in Singapore. Halstead SB, editor. PLoS Negl Trop Dis. 2011 Dec 20;5(12):e1426. doi: 10.1371/journal.pntd.0001426 22206028 PMC3243704

[24] National Environment Agency. Quarterly Dengue Surveillance Data [Internet]. [cited 2021 Sep 28]. https://www.nea.gov.sg/dengue-zika/dengue/quarterly-dengue-surveillance-data

[25] Ministry of Health Singapore. Weekly Infectious Diseases Bulletin [Internet]. https://www.moh.gov.sg/resources-statistics/infectious-disease-statistics/2018/weekly-infectious-diseases-bulletin

[26] Ministry of Health Singapore. Resources & Statistics [Internet]. https://www.moh.gov.sg/resources-statistics

[27] Standish K, Kuan G, Avilés W, Balmaseda A, Harris E. High Dengue Case Capture Rate in Four Years of a Cohort Study in Nicaragua Compared to National Surveillance Data. Halstead SB, editor. PLoS Negl Trop Dis. 2010 Mar 16;4(3):e633. doi: 10.1371/journal.pntd.0000633 20300515 PMC2838781

[28] Yew W, Ye T, Ang L, Ng L, Yap G, James L, et al. Seroepidemiology of dengue virus infection among adults in Singapore. Ann Acad Med Singap. 38:667–75. 19736569

[29] Egger JR, Coleman PG. Age and Clinical Dengue Illness. Emerg Infect Dis. 2007 Jun;13(6):924–7. doi: 10.3201/eid1306.070008 17553238 PMC2792851

[30] Porter KR, Beckett CG, Kosasih H, Tan RI, Alisjahbana B, Rudiman PIF, et al. Epidemiology of dengue and dengue hemorrhagic fever in a cohort of adults living in Bandung, West Java, Indonesia. Am J Trop Med Hyg. 2005 Jan 1;72(1):60–6. 15728868

[31] Beckett CG, Kosasih H, Faisal I, Nurhayati, Tan R, Widjaja S, et al. Early detection of dengue infections using cluster sampling around index cases. Am J Trop Med Hyg. 2005 Jun;72(6):777–82. 15967759

[32] Community Health Assist Scheme—Primary Care Clinics [Internet]. [cited 2021 Sep 28]. https://www.chas.sg/content.aspx?id=303

[33] Ang KL, Foo S. An exploratory study of eating patterns of Singapore children and teenagers. Health Educ. 2002 Oct;102(5):239–48.

[34] Committee on Ageing Issues. Report on the ageing population [Internet]. [cited 2021 Sep 28]. https://www.msf.gov.sg/publications/Documents/CAI_report.pdf

[35] World Health Organisation. WHO guide to identifying the economic consequences of disease and injury [Internet]. [cited 2021 Sep 28]. https://apps.who.int/iris/handle/10665/137037

[36] Land Transport Authority. Singapore Land Transport Statistics in Brief 2010 [Internet]. https://www.lta.gov.sg/content/dam/ltaweb/corp/PublicationsResearch/files/FactsandFigures/Statistics%20in%20Brief202015

[37] Haddix AC, Teutsch SM, Corso PS. Prevention effectiveness: a guide to decision analysis and economic evaluation. Oxford University Press; 2013.

[38] Singapore Ministry of Education. Education Statistics Digest 2010 [Internet]. 2010 [cited 2021 Sep 28]. https://perj.files.wordpress.com/2011/03/singaporeeducationstats2010.pdf

[39] Singapore Ministry of Education. Education Statistics Digest 2020 [Internet]. 2020 [cited 2021 Sep 28]. https://www.moe.gov.sg/about-us/publications/education-statistics-digest

[40] Household Income—Latest Data [Internet]. [cited 2021 Sep 28]. https://www.singstat.gov.sg/find-data/search-by-theme/households/household-income/latest-data

[41] Clark DV, Mammen MP, Nisalak A, Puthimethee V, Endy TP. Economic impact of dengue fever/dengue hemorrhagic fever in Thailand at the family and population levels. Am J Trop Med Hyg. 2005 Jun;72(6):786–91. 15964964

[42] Meltzer MI, Rigau-Pérez JG, Clark GG, Reiter P, Gubler DJ. Using disability-adjusted life years to assess the economic impact of dengue in Puerto Rico: 1984–1994. Am J Trop Med Hyg. 1998 Aug 1;59(2):265–71. doi: 10.4269/ajtmh.1998.59.265 9715944

[43] World Health Organisation. The global burden of disease: 2004 update. [Internet]. World Health Organisation; 2008 [cited 2021 Sep 28]. https://apps.who.int/iris/handle/10665/43942

[44] Gubler DJ, Meltzer M. Impact of Dengue/Dengue Hemorrhagic Fever on The Developing World. In: Advances in Virus Research [Internet]. Elsevier; 1999 [cited 2021 Sep 28]. p. 35–70. https://linkinghub.elsevier.com/retrieve/pii/S0065352708603425 doi: 10.1016/s0065-3527(08)60342-5 10582094

[45] Lum LCS, Suaya JA, Tan LH, Sah BK, Shepard DS. Quality of life of dengue patients. Am J Trop Med Hyg. 2008 Jun;78(6):862–7. 18541760

[46] Murray CJ. Quantifying the burden of disease: the technical basis for disability-adjusted life years. Bull World Health Organ. 1994;72(3):429–45. 8062401 PMC2486718

[47] Low JGH, Ong A, Tan LK, Chaterji S, Chow A, Lim WY, et al. The early clinical features of dengue in adults: challenges for early clinical diagnosis. PLoS Negl Trop Dis. 2011;5(5):e1191. doi: 10.1371/journal.pntd.0001191 21655307 PMC3104968

[48] Luz PM, Grinsztejn B, Galvani AP. Disability adjusted life years lost to dengue in Brazil. Trop Med Int Health TM IH. 2009 Feb;14(2):237–46. doi: 10.1111/j.1365-3156.2008.02203.x 19171013

[49] Death and Life Expectancy—Latest Data [Internet]. [cited 2021 Sep 28]. https://www.singstat.gov.sg/find-data/search-by-theme/population/death-and-life-expectancy/latest-data

[50] Ng LC, The Project Wolbachia–Singapore Consortium. Wolbachia-mediated sterility suppresses Aedes aegypti populations in the urban tropics [Internet]. Infectious Diseases (except HIV/AIDS); 2021 Jun [cited 2021 Sep 28]. http://medrxiv.org/lookup/doi/10.1101/2021.06.16.21257922

[51] World Health Organisation. WHO Guide for standardization of economic evaluations of immunization programmes: immunizations, vaccines, and biologicals [Internet]. [cited 2021 Sep 28]. https://apps.who.int/iris/bitstream/handle/10665/69981/WHO_IVB_08.14_eng.pdf?sequence=1&isAllowed=y

[52] Koopmanschap MA, Rutten FFH, van Ineveld BM, van Roijen L. The friction cost method for measuring indirect costs of disease. J Health Econ. 1995 Jun;14(2):171–89. doi: 10.1016/0167-6296(94)00044-5 10154656

[53] Shepard DS, Undurraga EA, Halasa YA, Stanaway JD. The global economic burden of dengue: a systematic analysis. Lancet Infect Dis. 2016 Aug;16(8):935–41. doi: 10.1016/S1473-3099(16)00146-8 27091092

[54] Tan Q, Hildon ZJ-L, Singh S, Jin J, Thein Tun Linn, Coker R, et al. Comparing patient and healthcare worker experiences during a dengue outbreak in Singapore: understanding the patient journey and the introduction of a point-of-care test (POCT) toward better care delivery. BMC Infect Dis [Internet]. 2017;17. https://www.proquest.com/scholarly-journals/comparing-patient-healthcare-worker-experiences/docview/1925198068/se-2?accountid=12691 doi: 10.1186/s12879-017-2580-9 28724363 PMC5517990

[55] Lim JT, Chew LZX, Choo ELW, Dickens BSL, Ong J, Aik J, et al. Increased Dengue Transmissions in Singapore Attributable to SARS-CoV-2 Social Distancing Measures. J Infect Dis. 2021 Feb 13;223(3):399–402. doi: 10.1093/infdis/jiaa619 33000172 PMC7543616

[56] Rajarethinam J, Ang L-W, Ong J, Ycasas J, Hapuarachchi HC, Yap G, et al. Dengue in Singapore from 2004 to 2016: Cyclical Epidemic Patterns Dominated by Serotypes 1 and 2. Am J Trop Med Hyg. 2018 Jul 5;99(1):204–10. doi: 10.4269/ajtmh.17-0819 29848407 PMC6085773

[57] Cook AR, Ng LC. Commentary: Uncovering the factors fueling record-high dengue cases in Singapore [Internet]. [cited 2021 Apr 2]. https://www.channelnewsasia.com/news/commentary/why-singapore-record-high-dengue-cases-covid-19-2020coronavirus-13160138

[58] Gubler DJ. Dengue, Urbanization and Globalization: The Unholy Trinity of the 21st Century. Trop Med Health. 2011;39(4SUPPLEMENT):S3–11. 10.2149/tmh.2011-S05 PMC3317603 22500131

[59] Lee LK, Thein TL, Kurukularatne C, Gan VC, Lye DC, Leo YS. Dengue knowledge, attitudes, and practices among primary care physicians in Singapore. Ann Acad Med Singapore. 2011 Dec;40(12):533–8. 22294064

[60] Daroudi R, Akbari Sari A, Nahvijou A, Faramarzi A. Cost per DALY averted in low, middle- and high-income countries: evidence from the global burden of disease study to estimate the cost-effectiveness thresholds. Cost Eff Resour Alloc CE. 2021 Feb 4;19(1):7. doi: 10.1186/s12962-021-00260-0 33541364 PMC7863358

[61] Population and Population Structure—Latest Data [Internet]. https://www.singstat.gov.sg/find-data/search-by-theme/population/population-and-population-structure/latest-data

[62] Utama IMS, Lukman N, Sukmawati DD, Alisjahbana B, Alam A, Murniati D, et al. Dengue viral infection in Indonesia: Epidemiology, diagnostic challenges, and mutations from an observational cohort study. Messer WB, editor. PLoS Negl Trop Dis. 2019 Oct 21;13(10):e0007785. doi: 10.1371/journal.pntd.0007785 31634352 PMC6822776

[63] Ang LW, Cutter J, James L, Goh KT. Seroprevalence of past dengue virus infection among children and adolescents in Singapore. J Med Virol. 2015 Dec;87(12):2159–62. doi: 10.1002/jmv.24287 26058712

[64] Low S-L, Lam S, Wong W-Y, Teo D, Ng L-C, Tan L-K. Dengue Seroprevalence of Healthy Adults in Singapore: Serosurvey Among Blood Donors, 2009. Am J Trop Med Hyg. 2015 Jul 8;93(1):40–5. doi: 10.4269/ajtmh.14-0671 26013376 PMC4497902

[65] Mendoza-Cano O, Hernandez-Suarez C, Trujillo X, Ochoa Diaz-Lopez H, Lugo-Radillo A, Espinoza-Gomez F, et al. Cost-Effectiveness of the Strategies to Reduce the Incidence of Dengue in Colima, México. Int J Environ Res Public Health. 2017 Aug 8;14(8):890. doi: 10.3390/ijerph14080890 28786919 PMC5580594

[66] Suaya JA, Shepard DS, Chang M-S, Caram M, Hoyer S, Socheat D, et al. Cost-effectiveness of annual targeted larviciding campaigns in Cambodia against the dengue vector Aedes aegypti: CE of annual targeted larviciding campaigns. Trop Med Int Health. 2007 Sep 14;12(9):1026–36. doi: 10.1111/j.1365-3156.2007.01889.x 17875014

[67] Luz PM, Vanni T, Medlock J, Paltiel AD, Galvani AP. Dengue vector control strategies in an urban setting: an economic modelling assessment. The Lancet. 2011 May;377(9778):1673–80. 10.1016/S0140-6736(11)60246-8 PMC3409589 21546076

[68] Fitzpatrick C, Haines A, Bangert M, Farlow A, Hemingway J, Velayudhan R. An economic evaluation of vector control in the age of a dengue vaccine. PLoS Negl Trop Dis. 2017 Aug;11(8):e0005785. doi: 10.1371/journal.pntd.0005785 28806786 PMC5573582

[69] Ho ZJM, Hapuarachchi HC, Barkham T, Chow A, Ng LC, Lee JMV, et al. Outbreak of Zika virus infection in Singapore: an epidemiological, entomological, virological, and clinical analysis. Lancet Infect Dis. 2017 Aug;17(8):813–21. doi: 10.1016/S1473-3099(17)30249-9 28527892

[70] Ang LW, Kam YW, Lin C, Krishnan PU, Tay J, Ng LC, et al. Seroprevalence of antibodies against chikungunya virus in Singapore resident adult population. Gubler DJ, editor. PLoS Negl Trop Dis. 2017 Dec 27;11(12):e0006163. doi: 10.1371/journal.pntd.0006163 29281644 PMC5760101

