# Supplementary material for: Economic impact of dengue in Singapore from 2010 to 2020 and the cost-effectiveness of Wolbachia interventions
Source: PLOS Glob Public Health. 2021 Oct 13;1(10):e0000024. doi: 10.1371/journal.pgph.0000024 (PMC10021432; doi:10.1371/journal.pgph.0000024)
Supplement: S1 Appendix — (PDF) [file pgph.0000024.s001.pdf]

S1 Appendix

July 21, 2021

1 Sensitivity analysis of parameters (Economic Costs)

	Constant symptomatic rates		Age dependent symptomatic rates	
25% increase in parameter	Human capital	Friction cost	Human capital	Friction cost
Death discount rate	1.032	0.459	1.016	2.254
Ambulatory expansion factor (Constant)	1.425	0.742	1.000	1.000
Ambulatory expansion factor (Age-dependent)	1.000	1.000	2.666	1.245
Hospital expansion factor	0.656	0.410	3.045	1.960
Elasticity of labour product	0.482	1.025	2.177	1.009
Transport cost	0.638	0.346	3.007	1.633
Household service lost	1.033	0.460	1.016	2.259
Proportion of elderly requiring caregiving	1.429	0.744	1.348	0.765
Proportion of children requiring caregiving	0.483	1.061	2.184	1.026
Days absent from ambulatory visit	1.217	0.545	1.223	2.719
Number of ambulatory visits	1.706	0.900	1.649	0.939

Table 1: Numbers represent the proportion increase in economic cost estimates for dengue from 2010 to 2020 from baseline levels given a 25% increase in the value of the respective parameter.

2 Sensitivity analysis of parameters (Health burdens)

	Age Disability Weight		Disease Disability Weight	
25% increase in parameter	Age Dependent EF	Constant EF	Age Dependent EF	Constant EF
D_{child}	1.009	1.977	1.387	2.760
D_{adult}	0.635	1.249	0.716	1.416
D_{df}	0.724	1.422	1.001	1.993
D_{dhf}	0.364	0.714	0.619	1.241
r	0.967	1.900	1.336	2.661
C	0.638	1.253	0.882	1.755
β	0.474	0.896	0.654	1.254
Duration of disability (Reported DF)	0.430	0.843	0.595	1.181
Duration of disability (Unreported DF)	1.002	1.969	1.385	2.758
Duration of disability (DHF)	0.511	1.003	0.705	1.405
Proportion DHF	0.724	1.422	1.000	1.992
Proportion hospitalized	0.364	0.714	0.502	1.000
Expansion factor (Constant)	1.000	1.969	1.000	2.758
Expansion factor (Age-dependent)	0.510	1.000	0.705	1.000
Expansion factor (Hospitalization)	0.372	0.723	0.511	1.009
Symptomatic rate	0.724	1.422	1.000	1.992

Table 2: Numbers represent the proportion increase in health cost estimates for dengue from 2010 to 2020 from baseline levels given a 25% increase in the value of the respective parameter. EF denotes expansion factor
